# A stochastic filtering approach to recover strain images from quasi-static ultrasound elastography

**DOI:** 10.1186/1475-925X-13-15

**Published:** 2014-02-12

**Authors:** Minhua Lu, Dan Wu, Wan-hua Lin, Weifang Li, Heye Zhang, WenHua Huang

**Affiliations:** 1 National-Reginoal Key Technology Engineering Laboratory for Medical Ultrasound, Guangdong Key Laboratory for Biomedical Measurements and Ultrasound Imaging, Department of Biomedical Engineering, School of Medicine, Shenzhen University, Shenzhen, China; 2 Key Lab for Health Informatics of the Chinese Academy of Sciences, Shenzhen Advanced Institutes of Technology, Chinese Academic of Sciences, Shenzhen, China; 3 Institute of Clinical Anatomy, Southern Medical University, Guangzhou, China

## Abstract

**Background:**

Model-based reconstruction algorithms have shown potentials over conventional strain-based methods in quasi-static elastographic image by using realistic finite element (FE) or bio-mechanical model constraints. However, it is still difficult to properly handle the discrepancies between the model constraint and ultrasound data, and the measurement noise.

**Methods:**

In this paper, we explore the usage of Kalman filtering algorithm for the estimation of strain imaging in quasi-static ultrasound elastography. The proposed strategy formulates the displacement distribution through biomechanical models, and the ultrasound-derived measurements through observation equations. Through this filtering strategy, the discrepancies are quantitatively modelled as one Gaussian white noise, and the measurement noise of ultrasound data is modelled as another independent Gaussian white noise. The optimal estimation of kinematic functions, i.e. the full displacement and velocity field, are computed through this Kalman filter. Then the strain images can be easily calculated from the estimated displacement field.

**Results:**

The accuracy and robustness of our proposed framework is first evaluated in synthetic data in controlled conditions, and the performance of this framework is then evaluated in the real data collected from elastography phantoms and patients with favourable results.

**Conclusions:**

The potential of our algorithm is to provide the distribution of mechanically meaningful strain under a proper biomechanical model constraint. We address the model-data discrepancy and measurement noise by introducing process noise and measurement noise in our framework, and then the mechanically meaningful strain is estimated through the Kalman filter in the minimum mean square error (MMSE) sense.

## Introduction

A tumour or a suspicious pathological growth is normally much stiffer than the background of normal soft tissue [1]. So when a mechanical compression or vibration is applied, the tumour deforms less than the surrounding tissue, i.e. the strain in the tumour is less than the surrounding tissue. Hence a strain image may, under particular simplifying assumptions, be interpreted as representative of the underlying Young’s modulus distribution. Ultrasound elastography [1] is a non-invasive technique in which stiffness or strain images of soft tissue are used to detect or classify tumours or cancers. Similar elastography techniques have been also developed using magnetic resonance imaging [2-4] and mammographic imaging [5]. As an emerging medical imaging modality, it has been broadly applied in the clinical applications, including improving the diagnostic accuracy of breast and prostate cancer [6-9], assessing plaque vulnerability [10-13], guiding minimally invasive therapy [14-16]. Currently, supersonic shear imaging (SSI) has become one cutting-edging technique for real-time visualisation of soft tissue mechanical properties. Using ultrasonic focused beams, SSI is able to remotely generate mechanical vibration sources radiating low-frequency, shear waves inside tissues, and then reconstruct the viscoelastic properties of tissue from the propagated shear waves [17]. Among different forms of ultrasound elastography, quasi-static ultrasound elastography is one popular technique because of its simplicity: two sets of radio-frequency (RF) echo frames are first collected before and after deformation caused by external compressions, and then the displacement field, strain field and even the distribution of tissular elasticity are reconstructed from RF signals using different approaches [18]. Numerous techniques have been proposed to solve the reconstruction problem of strain images for quasi-static ultrasound elastography in the last decades [19], but the estimation of strain images in ultrasound elastography still remains as a challenging researching topic at present because of its ill-posed nature [20].

The general procedure of quasi-static elastography is first to recover internal tissue displacements from two ultrasound radio-frequency (RF) frames before and after tissue deformation due to a quasi-static external compression, and then strain image and even mechanical properties distribution, such as Young’s modulus, can be computed from the displacement field under certain assumptions. So the quality of tissue motion estimation determines the performance of quasi-static ultrasound elastography. The estimation of tissue displacements is inherently a three-dimensional problem, which means the displacement vector components physically involve in all three directions (x, y, z axis) simultaneously and continuously. However, early methods only focus on axial displacement estimation. Among these techniques, time delay estimation (TDE) is an important and widely used displacement estimation method [1]. Typically, TDE method is to find the best-matching segment in the delayed RF signal for a specific segment in the reference RF signal by computing the maximum or minimum of a pattern matching function. Cross-correlation is mostly used as a pattern matching function in TDE method, and several other matching techniques have been also employed, such as correlation coefficient, hybrid sign correlations, sum absolute differences (SAD) and sum squared differences (SSD). TDE provides accurate estimation of axial displacement, but it is normally time-consuming. In the quasi-static elastography, the motion of tissue caused by the compression from the probe normally occurs in the corresponding two-dimensional scanning plane. However, the spatial resolution in the axial direction is much higher than that in the lateral direction in ultrasound imaging, that is why the estimation of the axial component of the motion has received the most attention [1,21]. Recently, quite a few approaches have been proposed to recover the lateral and shear strain fields (i.e. reconstruct the strain tensor), Poisson’s ratio and Young’s modulus [21], but their performance is still compromised by the low resolution of lateral displacements and the noise from RF data.

Both for the calculation of strain images and the reconstruction of elastic parameters, accurate estimation of tissue displacements is the first important step that will critically affect the image qualities. Therefore, different motion tracking techniques have been developed to recover tissue displacements during the past two decades [21]. Different beam-forming schemes have been proposed to improve the lateral resolution. For instance, using large beam steering angle can obtain better lateral displacement measurements, but this method can be only implemented by the phased array. Different post-processing techniques have also been applied to improve the quality of lateral displacement, such as iteratively interpolation along the lateral direction or local affine transformation. In another work, a two-dimensional displacement field is calculated using the analytic minimization (AM) of cost functions that incorporate both the similarity of the amplitudes of RF signals and the displacement continuity [21]. However, lateral displacement estimation is still of order of magnitude less accurate than the axial displacement estimation. Thus, lateral strain failed in identifying the ablation lesion in patient experiments [21]. Researchers have also tried to integrate bio-mechanical constraint to recover high-resolution lateral displacements from the noisy data. With the assumption of a constant Poisson’s ratio (0.49), i.e. based on the biomechanical constraint of tissue incompressibility [22], lateral displacements were recovered from axial-strain measurements using the least-square technique. However, the least-square technique in this tissue-incompressibility-assumption (TIA) method cannot perfectly eliminate measurement noise. In addition, errors will be introduced when the tissue incompressibility assumption is invalid. The recovery of strain and elasticity of tissue from the sparse displacement is one inverse problem [2,23,24], therefore, it is necessary to develop a robust framework with a more meaningful bio-mechanical constraint to recover a full displacement field from the ultrasonic measurements.

The rest of this paper is organised as follows: section Methodology describes the details of our elasticity reconstruction method. The linear FE model is first introduced, followed by the discussion about the integration of the stochastic state space strategy and the dynamic equation for the quasi-static ultrasound elastography. In section Experiments and results, the results obtained from the experiments using simulated data, real phantom data and in-vivo clinical data are presented and discussed respectively. In each set of data, our results are compared to the strain-based maps. As the ground truths are available in simulation and phantom study, the results generated by our strategy are compared to the ideal results. In the clinical experiment, the elastographic images are compared to CT images with lesions indicated by the doctor. Finally, the discussion of our framework, concluding remarks and future research endeavours are outlined in section Discussion and section Conclusion respectively.

## Methodology

### Linear elasticity

In order to construct a realistic, yet computationally feasible, analysis framework using the imaging data and other available physical measurements such as pressure induced by the probe, the structure and material of the biological tissue should be properly modelled. For computational simplicity, in our current 2D implementation, we adopt the linear isotropic continuum material for the quasi-static ultrasound, where the stress and strain relationship obeys the Hooke’s law. Hence, in our current two dimensional implementation the linear isotropic continuum material is adopted to describe the mechanical behaviour of the tissue in quasi-static elastography for the computational simplicity, and the relationship of the stress *σ* and the strain *ε* obeys the Hooke’s law [25]:

(1) σ=Sε

where *S* is the strain-stress matrix.

Let *u*(*x*,*y*) and *v*(*x*,*y*) be the displacement along the *x*- and *y*-axis of a point, the infinitesimal strain tensor of the point is:

(2) $$\varepsilon =\left[\begin{array}{c} \frac{\partial u}{\partial x}\\ \frac{\partial v}{\partial y}\\ \frac{\partial u}{\partial y}+\frac{\partial v}{\partial x} \end{array}\right]$$

Under the plane strain condition [25], strain-stress matrix *S* can be derived as:

(3) $$S=\frac{E}{\left(1+\nu )(1-2\nu \right)}\left[\begin{array}{ccc} 1-\nu & \nu & 0\\ \nu & 1-\nu & 0\\ 0 & 0 & \frac{1-2\nu }{2} \end{array}\right]$$

*S* is a material-dependent matrix, *E* stands for the Young’s modulus, and *ν* stands for the Poisson’s ratio. Here, the Young’s modulus *E* and the Poisson’s ratio *ν* are two material-specific parameters. This fact is quite clear from these relationships that the internal stress caused by the deformation is a function of the displacement vector and the material parameters. Ideally, the problem could be tackled in three dimension to avoid the through-plane motion effect, but in this paper we only study the two-dimensional problem because the image of quasi-static ultrasound elastography are two dimensional data collected by the linear array of ultrasonic probe. Further, more realistic biomechanical models can also be adopted into current framework in same way, but a linear material model is used to illustrate the fundamental ideas and rationales of our filtering estimation strategy to recover the distribution of strains for quasi-static ultrasound elastography.

### Stochastic finite element method

In the past decades, the deterministic finite element method has been able to provide an effective and convenient platform for bio-mechanical studies in the past decades [26,27]. However, it does not have the capability to process the uncertainties of material properties, and kinematic observations, i.e. measurements of the displacement. Especially the ultrasound imaging-derived data are usually corrupted by noises of various nature. It is thus necessary to develop a strategy, the stochastic finite element method (SFEM), which has been used widely for structural dynamic analysis in probability analysis frameworks [28,29]. In SFEM, structural material properties are described by random fields, possibly with known prior statistics.

In our implementation, a Delaunay triangulated finite element mesh is constructed at the first image frame before compression. An isoparametric formulation defined in a natural coordinate system is used, where, for tri-nodal linear element, the basis functions are linear functions of the nodal coordinates [25]. With mesh constructed, assuming that the material parameters *E* (Young’s modulus) and *ν* (Poisson’s ratio) are temporally constant but varying spatially, we can have the following dynamic governing equation [25]:

(4) $$M\ddot{U}+C\dot{U}+\text{KU}=R$$

where *U* is the displacement vector, and *R* is the load vector. Mass matrix *M* here is a known function of the material density and is temporally and spatially constant. Stiffness matrix *K* is a function of Young’s modulus and Poisson’s ratio determined by the material constitutive law [25]. Damping matrix *C* is frequency dependent, and we assumed the presence of small proportional Rayleigh damping and so *C* = *αM* + *βK* in our implementation, where *α* and *β* are the the mass- and stiffness-proportional Rayleigh damping weighting coefficients, respectively [25]. In practice it is difficult to determine the damping parameters because they are frequency dependent. Our assumption of Raleigh damping was based on the very low damping exhibited by biological tissues during quasi-static elastography, and fixed the two weighting coefficients at 1%.

One initialisation issue of Equation (4) is how to measure the external loading vector (**R**) during freehand elastography. Considering the object system dynamics embodied in Equation (4), if any knowledge of the displacement vector (**U**) is available, it can be used as essential boundary conditions to recover the motion parameters of all other nodes. The following experiments involving synthetic and real imaging data provided a set of displacements at nodal points of the boundary (e.g., axial displacements), and they are employed in the following fashion. Let **U**_*b*_ = *b* be known from the imaging data at selected sampling nodes of the boundary, then the additional constraining equation *μ***U**_*b*_ = *μb* is enforced on the system dynamics through

(5) $$M\ddot{U}+C\dot{U}+KU+\mu U_{b}=R+\mu b$$

where weighting coefficient *μ* depends on the confidence of each displacement, with large *μ* values (1 × 10^4^ in this study) indicating highly trustworthy data points and small *μ* values for others. In this way it remains possible to describe the boundary condition without measuring the external force during freehand elastography. More details of this enforcement of boundary condition can be found elsewhere [25].

### State space representation

In order to employ our filtering strategy to integrate bio-mechanical model, the dynamics equation (Equation (4)) needs to be transformed into a state-space representation of the continuous-time linear stochastic system. First the kinematic vector *x*(*k*) and the material parameter vector *θ* are defined as:

(6) $$x(t)=\left[\begin{array}{c} U(t)\\ \dot{U}(t) \end{array}\right]$$

(7) $$\theta =\left[\begin{array}{c} E\\ \nu \end{array}\right]$$

where the kinematic vector *x*(*t*) is consisted of displacement *U*(*t*) and velocity $\dot{U}(t)$ information, and the material parameter vector *θ* is consisted of Young’s modulus *E* and Poisson’s ratio *ν*. In general, tumour inclusion or tissue blocked from its blood nutrients is stiffer than normal tissue, which mostly reflects in the variation of *E*. Since benign and cancerous tumours usually have distinguishing elastic properties, i.e. different Young’s modulus value, the value of Poisson’s ratio can be fixed in the following implementation. For example, as one of incompressible materials, the Poisson’s ratio of tissue can be set close to 0.5.

The state space representation of Equation (4) becomes

(8) $$\dot{x}(t)=A_{c}x(t)+B_{c}W(t)+n(t)$$

where *n*(*t*) is the process noise and it is white noise (*E*[ *n*(*t*)] = 0; *E*[ *n*(*t*)*n*(*s*)^′^] = *Q*_*n*_(*t*)*δ*_*ts*_, where *Q*_*n*_ is the process noise covariance). The system matrices *A*_*c*_, *B*_*c*_ and input forces *W*(*t*) are:

$$\begin{array}{ll} \;A_{c} & =\left[\begin{array}{cc} 0 & I\\ -M^{-1}K & -M^{-1}C \end{array}\right]\\ \;B_{c} & =\left[\begin{array}{cc} 0 & 0\\ 0 & -M^{-1} \end{array}\right]\\ \;W(t) & =\left[\begin{array}{c} 0\\ R(t) \end{array}\right] \end{array}$$

An associated measurement equation, which describes the observations *y*(*t*) provided by the ultrasonic RF data, can be expressed in the form:

(9) y(t)=Hx(t)+e(t)

where *e*(*t*) is the measurement noise and it is white noise (*E*[*e*(*t*)] = 0; *E*[*e*(*t*)*e*(*s*)^′^] = *R*_*e*_(*t*)*δ*_*ts*_, and *R*_*e*_ is the measurement noise covariance), independent of *n*(*t*). *H* is the measurement matrix which should be specified by the relation between state vector *x*(*t*) and measurement vector *y*(*t*).

In order to run the dynamic equation (Equation (4)) in computer, it should be transformed into a discrete state-space equation, typically seen in control and estimation literature [30,31]. We discretize Equation (8) and (9) over a constant time interval *T*. Since the interval *T* is always a known constant, we can replace *kT* with *k* in following equations

(10) x(k+1)=Ax(k)+Bw(k)+n(k),

where

(11) $$\begin{array}{l} A=e^{A_{c}T}, \end{array}$$

(12) $$\begin{array}{l} B=A_{c}^{-1}(e^{A_{c}T}-I)B_{c} \end{array}$$

The associated measurement equation can be:

(13) y(k)=Dx(k)+e(k)

where *y*(*k*) is the measurement vector contained the displacement extracted from ultrasound RF data. In most quasi-static elastography, only theaxial component of displacement vector is extracted. Therefore, the corresponding place of *D* will be set to 1 or 0 according to the available measurement data. *n*(*k*) and *e*(*k*) are process noise and the measurement noise respectively.

## Experiments and results

Our filtering framework is validated in simulated data, real phantom data and clinical data respectively. All the programs in the following experiments are coded in Matlab 2009a (MathWorks Corporation, USA) and run in a desktop computer with a core 2 intel CPU and 12 G memory.

### Simulated data

**Figure 1:**
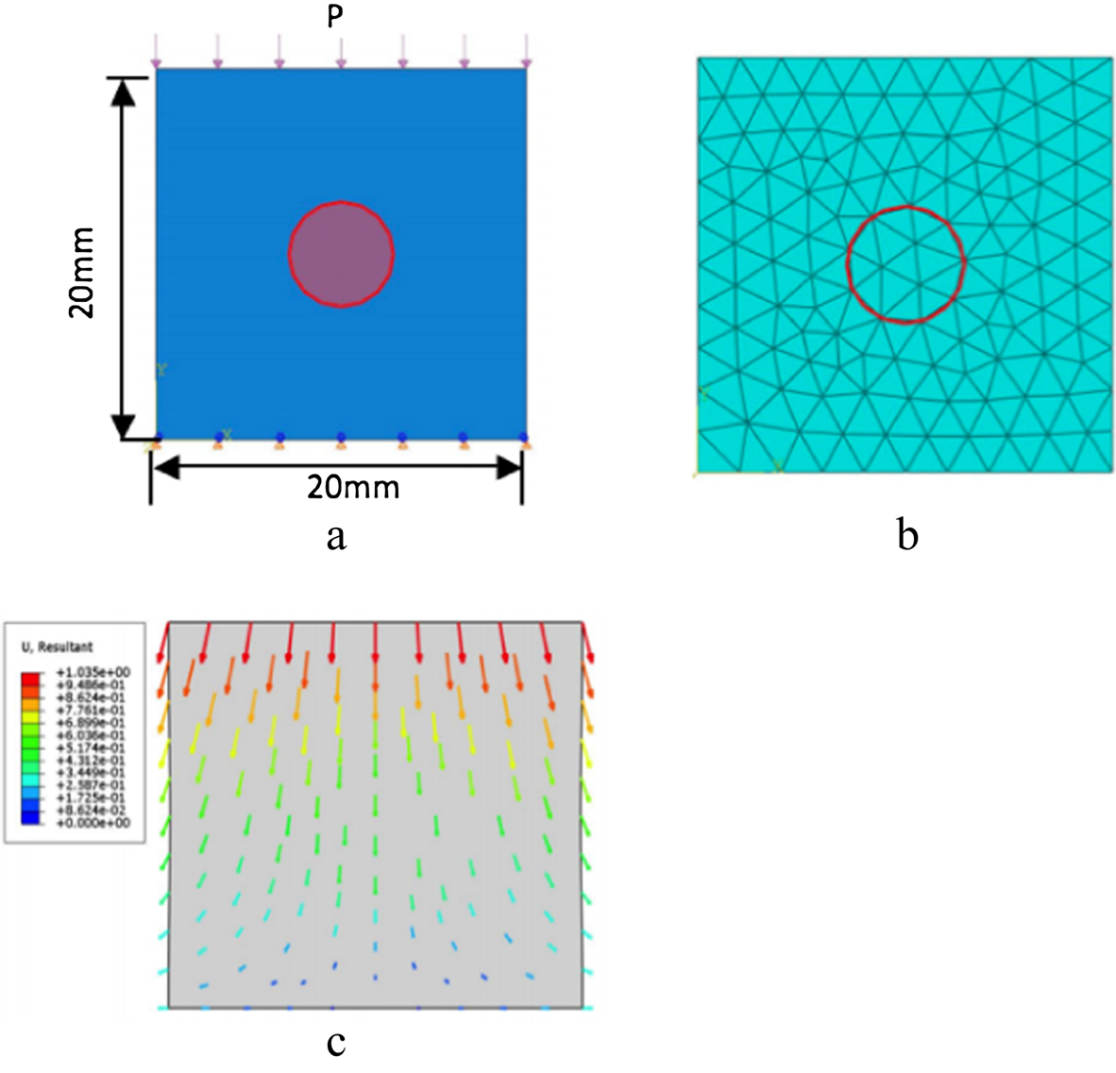
**Setup of phantom model.** **(a)** the model with circular inclusion; **(b)** the finite element mesh; **(c)** the direction of moved nodes.

**Figure 2:**
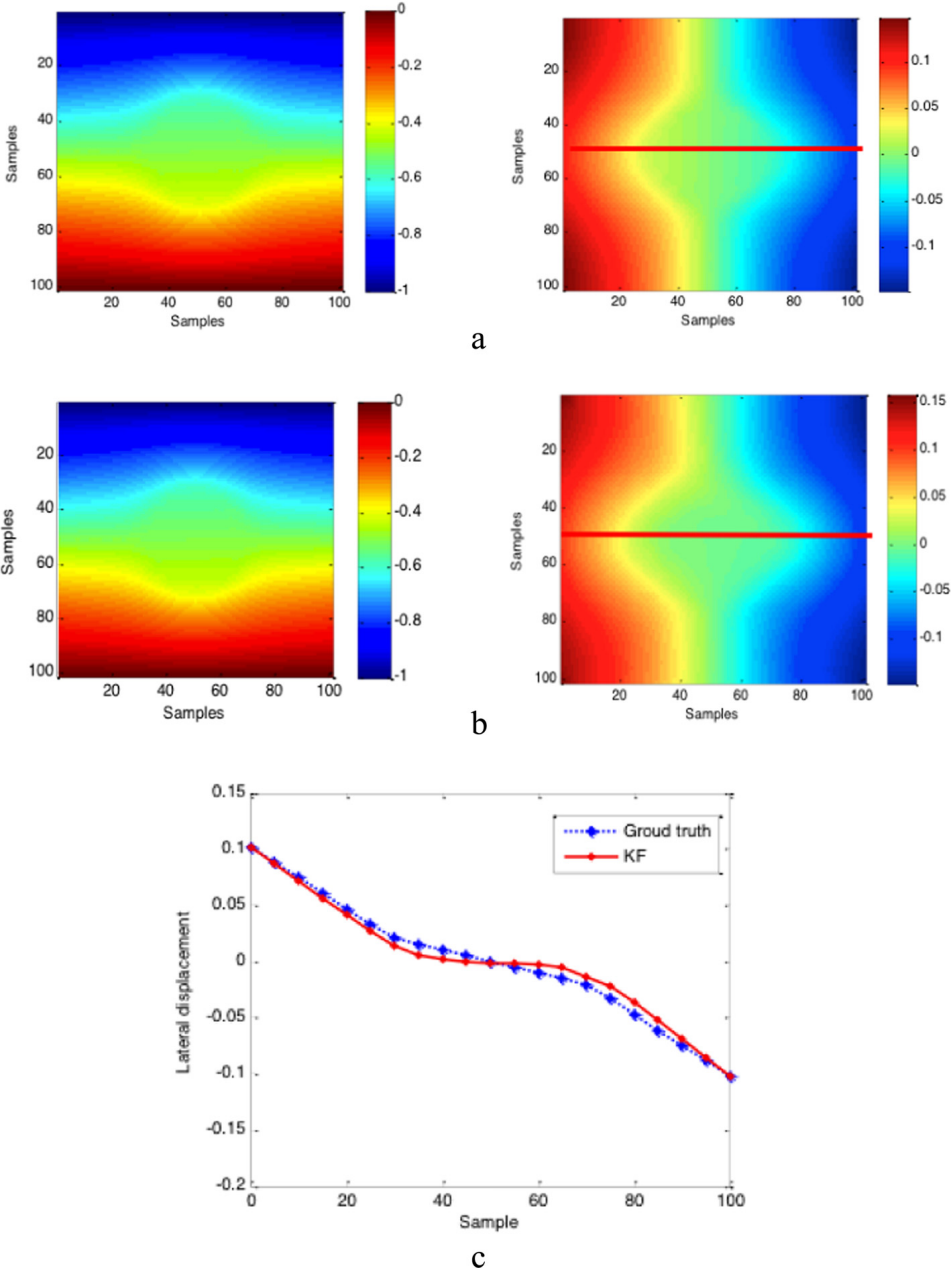
**Comparison between recovered displacement field and true displacement field.** True displacement field and recovered displacement field: **(a)** ground truth of the axial displacement (left) and lateral displacement (right); **(b)** the estimated results: axial displacement (left) and lateral displacement (right); **(c)** the comparison of lateral displacement profile at the same depth which is marked by the colourful lines.

**Figure 3:**
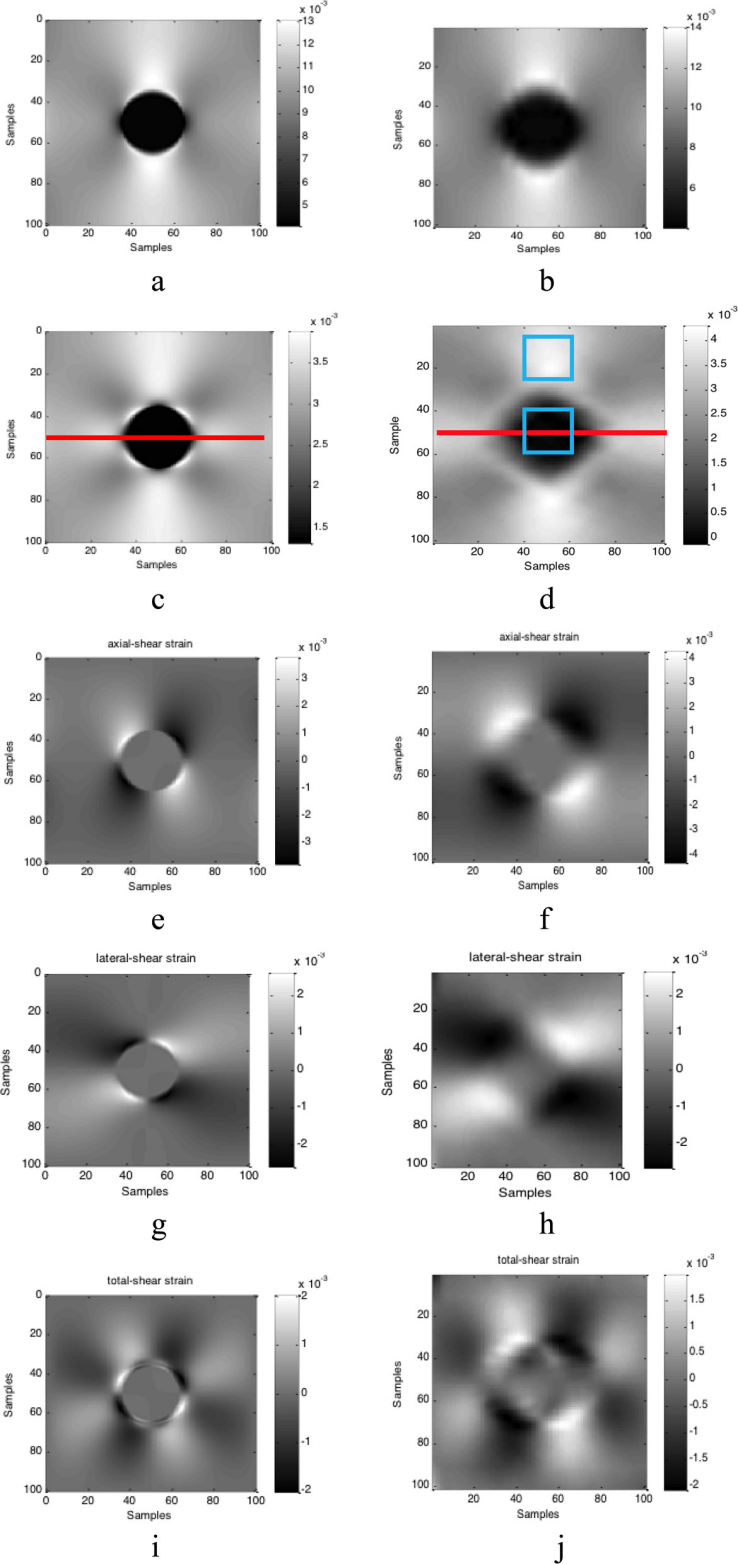
**Comparison between recovered strain field and true strain field.** True strain field: **(a)** axial strain, **(c)** lateral strain, **(e)** axial-shear strain, **(g)** lateral-shear strain, and **(h)** shear strain; recovered strain field: **(b)** axial strain, **(d)** lateral strain, **(f)** axial-shear strain, **(h)** lateral-shear strain, and **(j)** shear strain.

**Figure 4:**
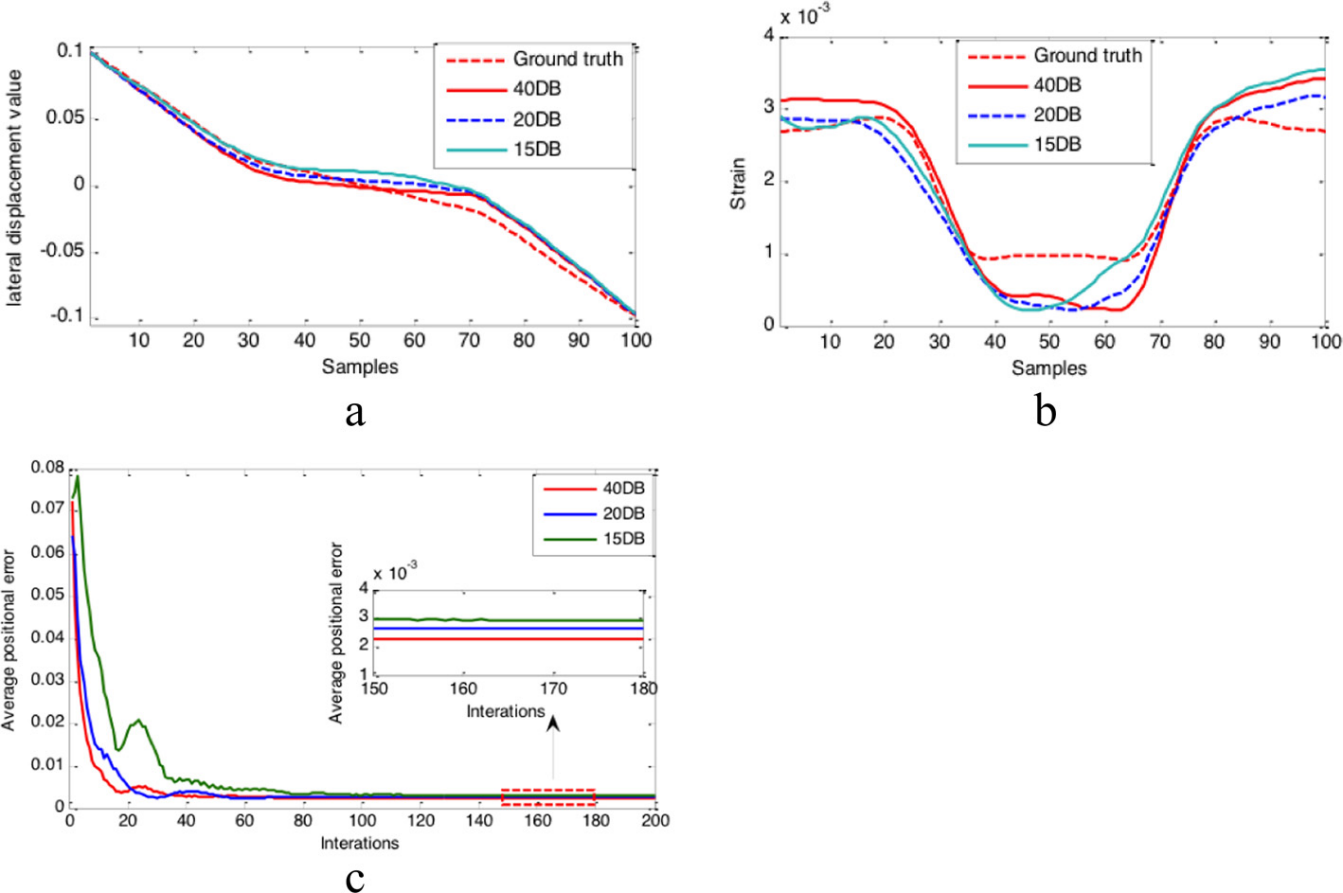
**Performance evaluation under different external noise conditions.** Comparison of estimated results under different noise conditions (40 dB, 20 dB, 15 dB): **(a)** estimated lateral displacement profiles at the same depth which is marked by the red lines in Figure 3; **(b)** estimated strain profiles at the same depth which is marked by the red lines in Figure 3**(c)** converge curves of average positional error, and the small segments of the curves indicated by the red colour rectangular area are zoomed in displayed in the small panel.

**Figure 5:**
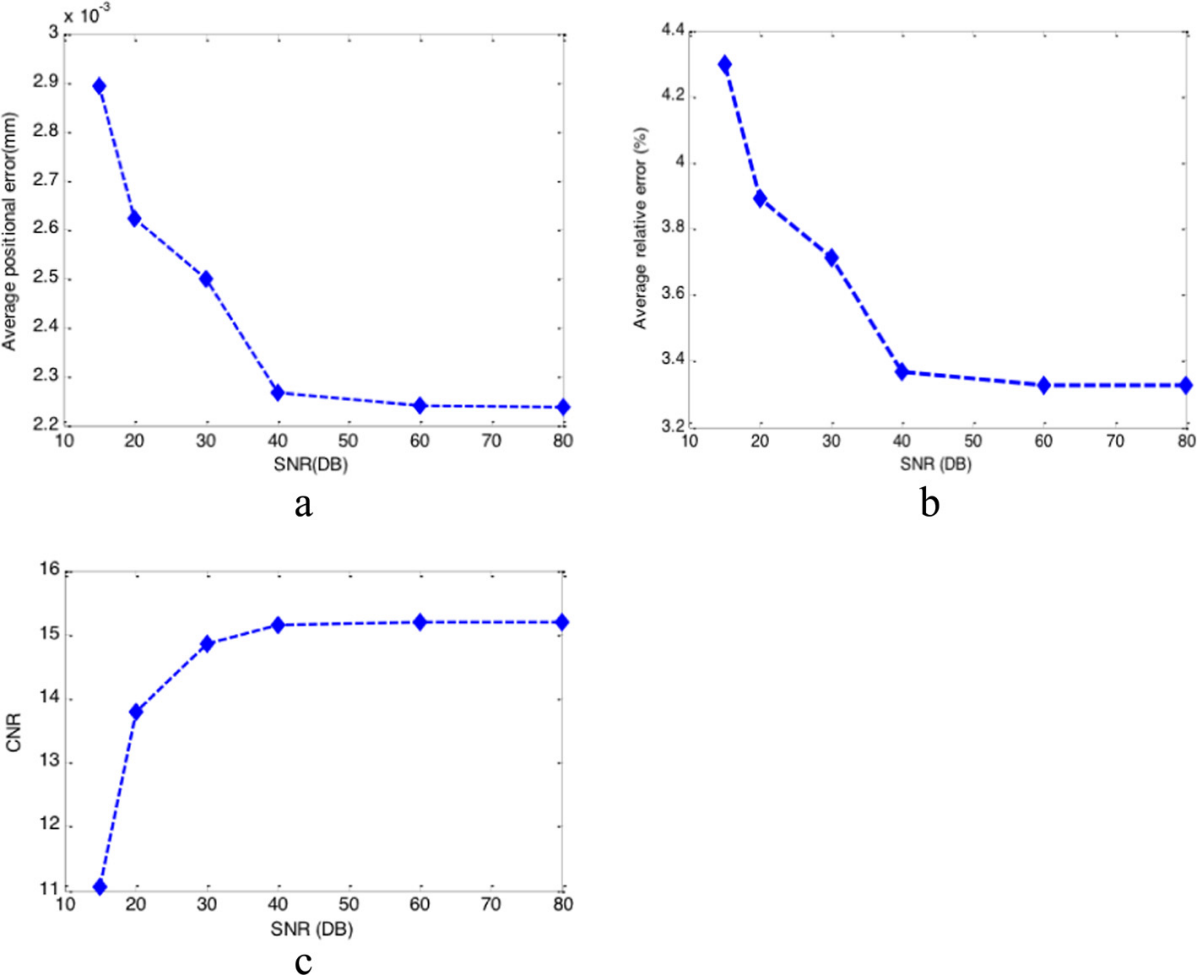
**Tolerance of external noise.** Three factors’ tolerance of external noise conditions (from 15 db to 80 db): **(a)** average position error; **(b)** average relative error; **(c)** CNR (the used areas are indicated by blue lines in Figure 3(d)).

ABAQUS (DS Simulia Corporation, USA) software is used for generating simulation data, and our phantom model is shown in Figure 1. Because the displacements are collected by the ultrasound probe along axial direction and the lateral displacement is much less accurate than axial displacement, we only use the axial displacements as input data and estimate the full displacement field through our method. In this model, a 20×20 mm rectangular object (*E*_*b*_ = 25 kPa and *ν*_*b*_ = 0.49) is built as the background. A 4 mm hard circular inclusion with different elastic property (*E*_*t*_ = 80 kPa and *ν*_*t*_ = 0.49) is included in the centre of the model, as the target. The top side of the object is compressed to move 1.0 mm down along the vertical direction with a deformation ratio 5% and the bottom side is fixed. After the rectangular with circular is divided into a triangular mesh and the mechanical deformation is calculated by ABAQUS, the Gaussian noise is added into the axial displacements for generating simulated the measurements. The lateral displacement is recovered by our proposed Kalman filter method, then a linear interpolation function is applied to smooth the displacement image. The least square method is used to calculate the strain field. We design two experiments on the simulated data in different conditions: 

1. Since we have the ground truth of simulated measurements, the first experiment is designed to examine the influence of measurement noise. In order to evaluate the performance quantitatively, we define the following two factors, the lateral displacement relative error (DRE) and contrast-to-noise ratio (CNR):

(14) $$\begin{array}{l} \text{DRE}=\frac{\bar{e}}{\bar{d}} \end{array}$$

(15) $$\begin{array}{l} \text{CNR}=\sqrt{\frac{2{\left(\bar{s_{b}}-\bar{s_{t}}\right)}^{2}}{\sigma_{b}^{2}+\sigma_{t}^{2}}} \end{array}$$

where $\bar{e}$ is the average nodal displacement error, $\bar{d}$ is the average nodal displacement value, $\bar{s_{t}}$ and $\bar{s_{b}}$ are the spatial strain average of the target and background, $\sigma_{t}^{2}$ and $\sigma_{b}^{2}$ are the spatial strain variance of the target and background. Figure 2 and Figure 3 show the lateral displacement and strains estimated results when the axial displacement is added by the Gaussian noise (SNR = 40 dB). Figure 4 and Figure 5 show the estimated results in different noise conditions (40 db, 20 db, 15 db). Table 1 shows the comparison of lateral displacement errors and CNR under different measurement noise levels.

**Table 1:** Comparison of lateral displacement errors and CNR under different measurement noise levels

**SNR**	**80 dB**	**60 dB**	**40 dB**	**30 dB**	**20 dB**	**15 dB**
Average positional error (× 10^-3^ mm)	2.2411	2.2412	2.2679	2.5023	2.6250	2.8952
Average relative error (%)	3.3270	3.3271	3.3667	3.7147	3.8922	4.2980
CNR	15.1921	15.1910	15.1561	14.8592	13.7931	11.0543

**Figure 6:**
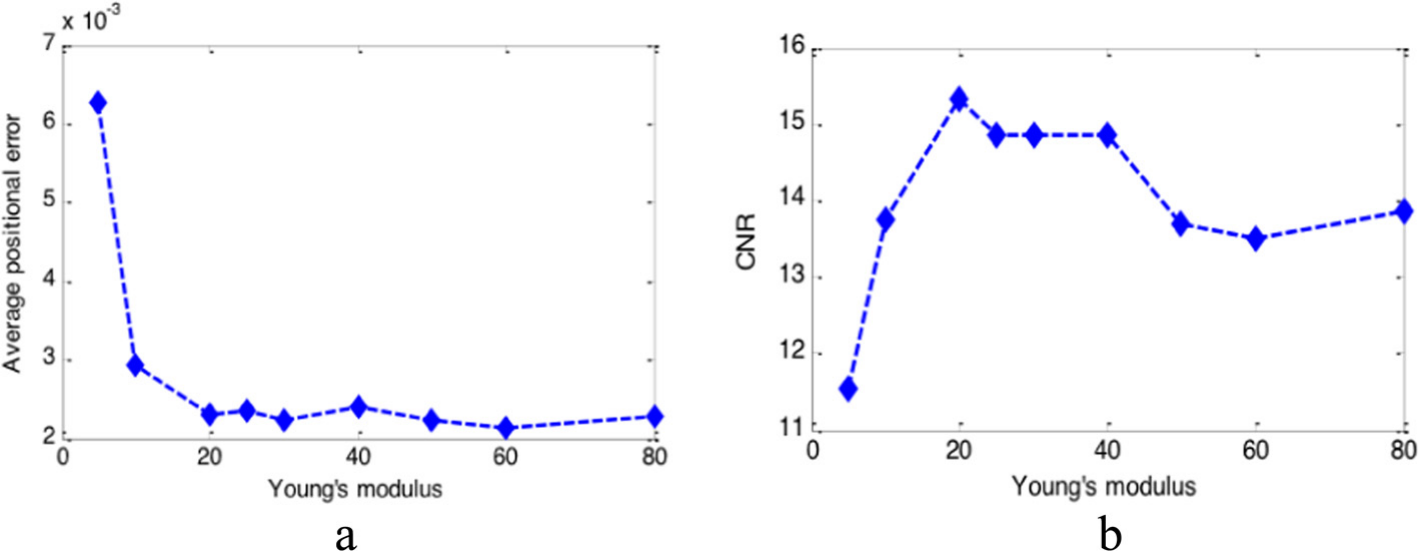
**Tolerance of Young’s modulus.** Estimated results using increased Young’s modulus: **(a)** average position error and **(b)** CNR (the used areas are indicated by blue lines in Figure 3(d)).

2. The second experiment is designed to evaluate the effect of initialisation of the model elastic property. In our method Young’s modulus distribution is assumed to be homogeneous when the whole displacement field is estimated. Figure 6 and Table 2 show the effect of Young’s modulus in lateral displacement estimation. The performance of our method is acceptable when Young’s modulus is greater than 20 kPa, but the CNR is in a higher level only from 20 kPa to 40 kPa.

**Table 2:** Comparison of lateral displacement errors and CNR using different Young’s modulus

**Young’s modulus (kPa)**	**5**	**10**	**20**	**30**	**40**	**50**	**60**	**80**
Average positional error (× 10^-3^ mm)	6.2704	2.9314	2.3100	2.2253	2.3947	2.2381	2.1240	2.2818
Average relative error (%)	9.3085	4.3518	3.4291	3.3036	3.5550	3.3226	3.1531	3.3874
CNR	11.526	13.750	15.322	14.842	14.860	13.685	13.506	13.860

### Real phantom data

**Figure 7:**
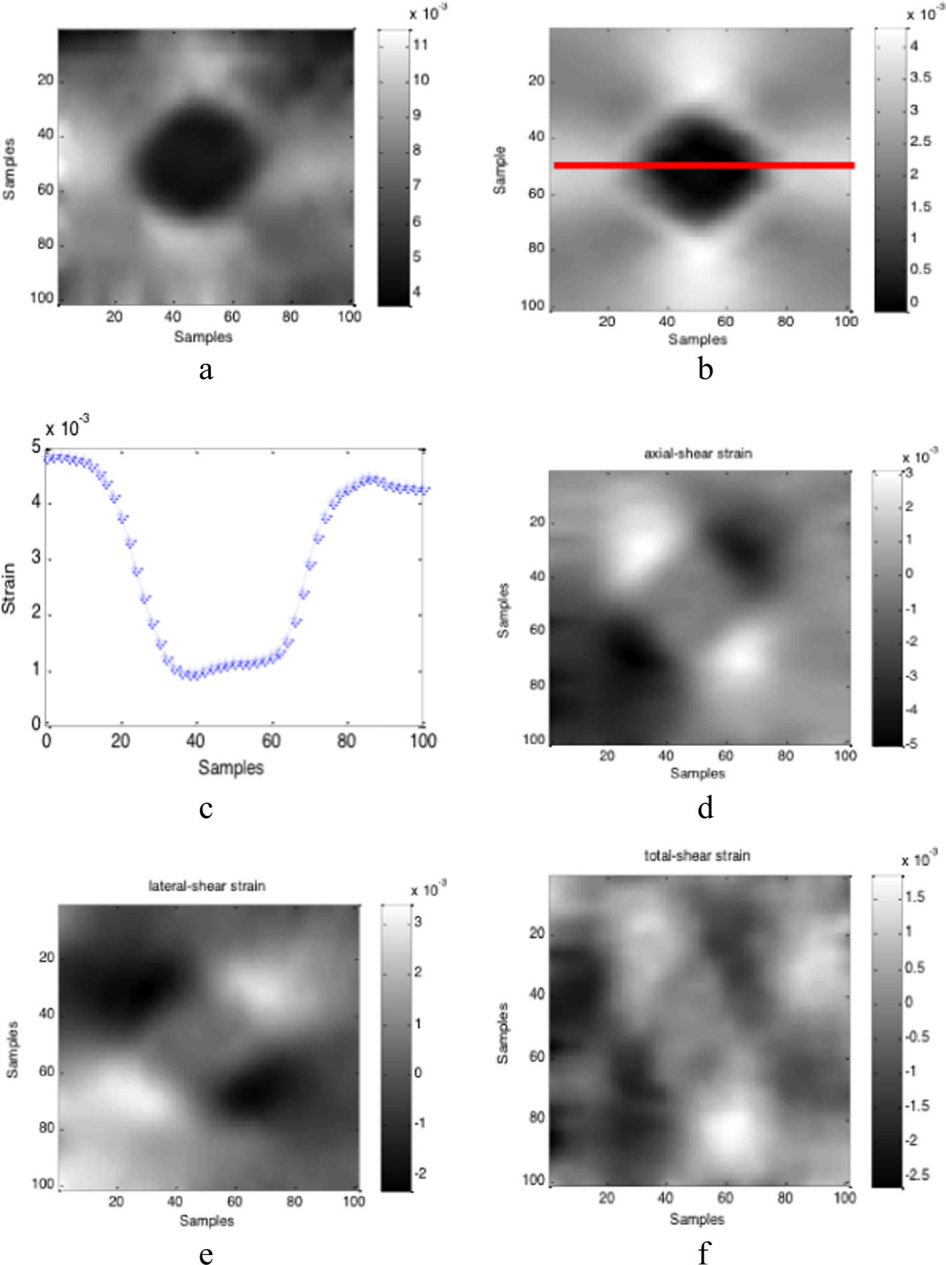
**Experiment on phantom data.** Estimated strain field using phantom data: **(a)** axial strain, **(b)** lateral strain, **(c)** lateral strain profile at the same depth which is marked by the red line in **(b)**, **(d)** axial-shear strain, **(e)** lateral-shear strain, and **(f)** shear strain.

**Figure 8:**
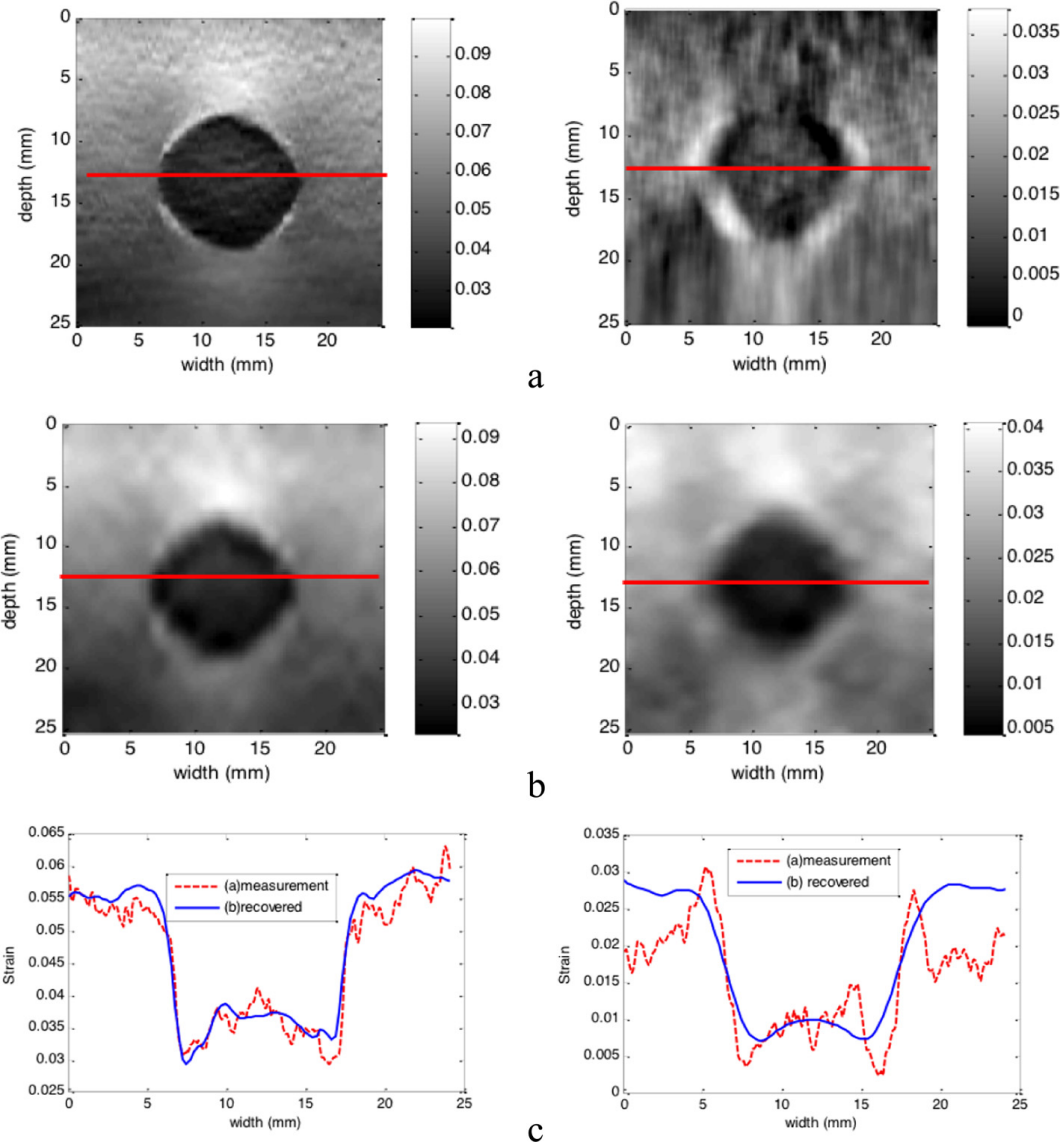
**Comparison of our method and JHU method.** **(a)** axial and lateral strain images (left to right) generated by JHU method; **(b)** axial and lateral strain images (left to right) generated by our model-based framework; **(c)** Comparison of axial strain profiles (left) and lateral strain profiles (right) at the same depth which is marked by the red lines.

Two sets of phantom data are used to evaluate the performance of our method. The first set of phantom data is collected by us in one Elasticity QA Phantom (Model 049, CIRS Inc. USA) using a PC-based Ultrasonix RP ultrasound machine (Ultrasonix Medical Corporation, Burnaby, BC, Canada). One phase-shift method with prior estimates [32] is used to estimate the axial displacements from the RF signals, and the full displacement field is recovered from the axial displacement measurements. And then all the strain images are displayed in Figure 7. We also compare our method to the method developed by Johns Hopkins University (JHU) [21]. In this comparison, the second set of phantom data is provided by JHU and it is available online [21]. Figure 8 shows the recovered strain images from both methods and the strain profiles in the same depth.

### Clinical data

**Figure 9:**
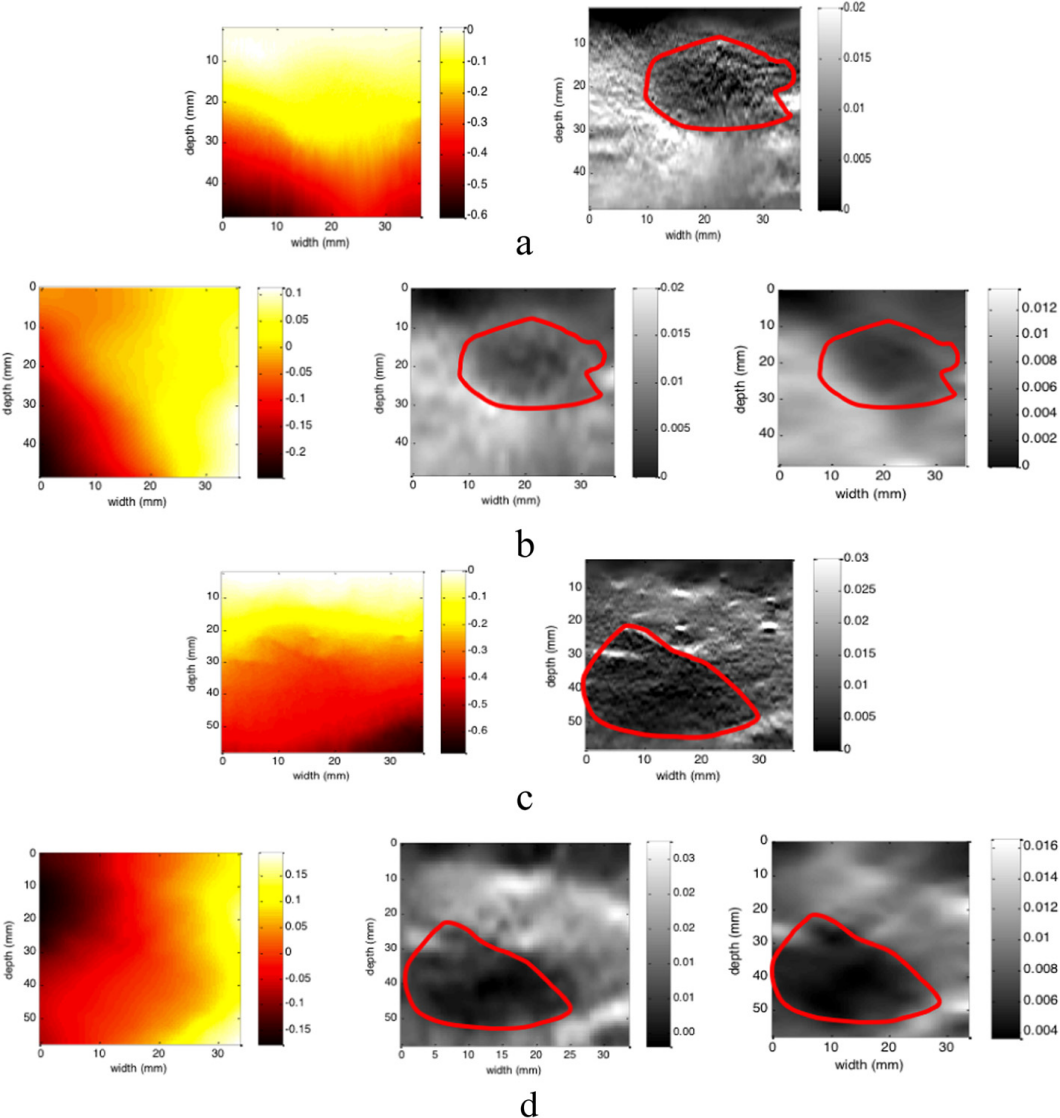
**Experiment on clinical data.** Patient 1: **(a)** axial displacement and strain images (left to right) calculated from JHU’s displacement results; **(b)** lateral displacement, axial strain and lateral strain images (left to right) calculated from displacements which are estimated by our method. Patient 2: **(c)** axial displacement and strain images (left to right) calculated from JHU’s displacement results; **(d)** lateral displacement, axial strain and lateral strain images (left to right) calculated from displacements which are estimated by our method.

The ultrasound experiments were performed with the approval of the Health Science Research Ethics Committee of JHU. The participants were provided written informed consent before beginning the experiment. Three sets of patients’ ultrasonic RF data are provided online in [21], and those data are collected in the patients undergoing open surgical RF thermal ablation for liver cancer enrolled between February 06, 2008 and July 28, 2009. The details of these patients’ clinical status have been extensively discussed in [21]. All the patients’ data are processed in the same way as the way for real phantom data. Since the axial and lateral displacement measurements have been provided in [21], the extraction of displacement is not necessary in this experiment. Figure 9 shows patients’ CT images scanned after RF ablation, strain images and our estimated results respectively.

## Discussion

Our model-based stochastic filtering strategy is able to provide a a high-resolution displacement field (in both axial and lateral directions) in 2D elastography. In the following context the robustness, initialisation and computational cost of our filtering framework are discussed respectively.

### Robustness of our filtering strategy

The kernel of our proposed approach is the Kalman filtering algorithm, therefore the tolerance of external noise is the one key performance factor of our filtering strategy. Our filtering framework can recursively absorb sparse measurements under the biomechanical-model-based constraint, and finally calculate physically meaningful optimal estimates, which is a full and smoother displacement field. In order to examine the robustness of our filtering strategy, we have designed several experiments to test the tolerance of the noise in our method.The tolerance of the proposed filtering strategy to external noise was evaluated by adding different levels of measurement noise to the simulated measurement. Our filtering framework was still able to recover the displacement/strain fields with a similar quality from measurements containing different levels of measurement noise after certain number of iterations, as indicated by the three pictures in Figure 4. But if we examine the tolerance of noise in much finer scale, we can see the quality of estimated displacement/strain field will not change when SNR is greater than 40 db as shown in Figure 5. This fact can be also verified by quantitative measures in Table 1.

### Initialisation issues

The number of iterations and the initialisation of biomechanical-model constraint (Young’s modulus and Poisson’s ratio) are important to our filtering strategy. In order to generate a displacement/strain image of high quality, we simply performed 200 iterations of the filtering procedures as show in Figure 4(c). Since most biological tissues are incompressible materials, Poisson’s ratio used in our filtering strategy can be initialised to 0.49. When Young’s modulus is initialised greater than 20 kPa, the average position error will not increase anymore (see Figure 6(a) and Table 2), but a better CNR can only be obtained when Young’s modulus is from 20 kPa to 40 kPa (see Figure 6(b)). Therefore, the initial value of Young’s modulus should be in the range from 20 kPa to 40 kPa.

### Computational cost

The computational cost of our method is the high in current implement scheme. In all the experiments performed in this study we can only handle measurements on 21 × 21 sample nodes. Although the spatial resolution of the recovered strain image is actually limited by the number of nodes, the quality of the estimated strain images generated by our model-based filtering strategy is not yet compromised. Furthermore, we can take advantage from advanced computing power technologies, such as GPU, to accelerate our filtering strategy in future study.

## Conclusion

In this paper, we have developed a bio-mechanically constrained filtering framework to extract a full displacement field from the measurements derived from ultrasound RF signals collected before and after deformation. Then, the strain tensor can be reconstructed from the full displacement field. The proposed framework have been validated by a series of experiments. However, the spatial resolution of recovered displacement field and strain images are restricted by the number of finite element nodes. In the future works, more efforts should be done on computational complexity reduction in order to increase spatial resolution.

## Abbreviations

MMSE: Minimum mean square error; RF: Radio frequency; SFEM: Stochastic finite element method; FEM: Finite element method; FE: Finite element; TIA: Tissue-incompressibility-assumption; SNR: Signal-to-noise ratio; DRE: Displacement relative error; CNR: Contrast-to-noise ratio; JHU: Johns Hopkins University.

## Competing interests

The authors declare that they have no competing interests.
